# Development and pharmacokinetic evaluation of granisetron hydrochloride buccal tablets for optimized chemotherapy-induced nausea and vomiting (CINV) relief

**DOI:** 10.3389/fphar.2026.1731072

**Published:** 2026-03-26

**Authors:** Chandrashekar Thalluri, Sarad Pawar Naik Bukke, Malliukarjun Vasam, Jharna Medhi, Ananda Kumar Chettupalli, Bharath Kumar Mamilla Mugaiahgari, Bayapa Reddy Narapureddy, Vinod Kumar Yata, Ravi Chander Thatipelli

**Affiliations:** 1 Faculty of Pharmaceutical Science, Assam down town University (AdtU), Guwahati, Assam, India; 2 Department of Pharmaceutics and Pharmaceutical Technology, Kampala International University, Western Campus, Kampala, Uganda; 3 Department of Pharmaceutics, Omega College of Pharmacy, Osmania University, Edulabad, Hyderabad, Telangana, India; 4 Department of Pharmacy, School of Medical and Allied Sciences, Galgotias University, Noida, Uttar Pradesh, India; 5 Department of Emergency Medical Services, College of Applied Medical Sciences, King Khalid University, Abha, Saudi Arabia; 6 Department of Public Health, College of Applied Medical Sciences, King Khalid University, Abha, Saudi Arabia; 7 Department of Pharmacology, School of Allied and Healthcare Sciences, Malla Reddy University, Hyderabad, Telangana, India; 8 Department of Pharmaceutics, Vaagdevi Pharmacy College, Warangal, Telangana, India

**Keywords:** chemotherapy-induced nausea and vomiting (CINV), DD solver and controlled release, granisetron hydrochloride, mucoadhesive polymers, response surface methodology

## Abstract

**Introduction:**

Chemotherapy-induced nausea and vomiting (CINV) limits treatment adherence; this study aimed to develop optimized Granisetron hydrochloride buccal tablets to improve therapeutic performance and bioavailability.

**Methods:**

Tablets were prepared by direct compression and optimized using response surface methodology with HPMC K4M (X_1_) and carbomer 934P (X_2_). Compatibility was assessed by FT-IR and DSC, while mucoadhesion, t_50_, drug release, kinetics, and pharmacokinetics were evaluated.

**Results:**

No drug–excipient interactions were observed. The optimized formulation (F3; 50 mg HPMC K4M and 15 mg carbomer 934P) showed adequate mucoadhesion (8.25 ± 0.12 g), prolonged release (t_50_ ≈ 323 ± 0.35 min), and 70.23% ± 2.14% release at 8 h. Release followed the korsmeyer–peppas model, indicating anomalous transport. Pharmacokinetic studies showed significantly higher C_max_ and AUC than oral tablets (*p* < *0.04*).

**Discussion:**

The optimized buccal system provides controlled release and enhanced pharmacokinetic performance, suggesting a promising alternative approach for effective CINV management.

## Graphical Abstract

**Figure:**
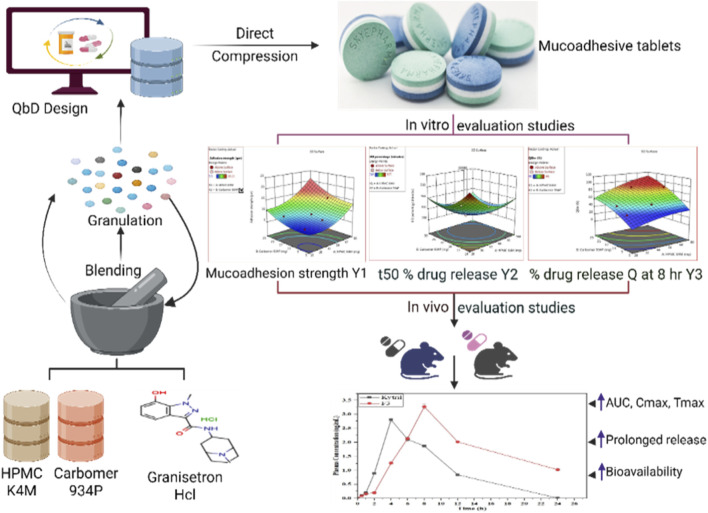

## 1 Introduction

Chemotherapy has long been an important therapy for a variety of cancers. By attacking quickly-dividing cells, it has changed how we treat cancer (Kaur et al., 2023; Raghani et al., 2024). Chemotherapy is effective, but it is also associated with many side effects that are distressing and impact a patient’s quality of life (Brianna and Lee, 2023; Ingole et al., 2024). Of all the side effects associated with chemotherapy, nausea and vomiting due to chemotherapy (CINV) is the most severe, as it represents a major clinical concern, as well as significant discomfort to the patient (Kaur et al., 2022). The CINV experience is influenced by the interaction of chemotherapy drugs with the central nervous system, gastrointestinal system, and outside receptors, making it a complicated, multi-faceted experience (Gupta et al., 2021). Neurotransmitters, such as serotonin, play a major role in triggering the brain’s vomiting center, resulting in vomiting and nausea (Vardy et al., 2022). Consequently, patients’ quality of life deteriorates due to the physical effects of nausea and vomiting, in addition to the mental distress related to treatment non-compliance and the overall negative impact on the therapeutic experience (Lewandowska et al., 2020). In most instances, CINV is treated with the use of antiemetics; the most well-known and commonly used class of antiemetics contains 5-HT_3_ receptor antagonists. Granisetron hydrochloride is one of the most popular 5-HT_3_ receptor antagonists available (Spartinou et al., 2017; Clark-Snow et al., 2018). Although these medications are effective in providing symptom relief from CINV, they do present unique challenges (Lorusso et al., 2020). Both oral and IV routes of administration have variable levels of absorption delaying their onset of action and dose-dependent adverse effects (Aapro, 2018). Additionally, repeating doses increases the challenge of patient non-compliance due to the long duration of chemotherapy (Rashidi et al., 2024). It is important to note that managing CINV is important not only for the comfort of patients, but also for the complete success of cancer treatment. According to Jacobs et al. (2019) and Aapro et al. (2021), when patients have less nausea and vomit less as a result of their medication, they are more likely to follow their doctor’s recommended chemotherapy plan; therefore, patients will receive maximum benefit from their chemotherapy and hence obtain the best possible therapy. Thus, enhancing the treatment of CINV not only directly increases the quality of life of cancer patients but also indirectly supports the success of various methods for treating the disease (Clemons, 2018; Navari, 2016). We are conducting studies on buccal tablets and their ability to control the buccal release of Granisetron hydrochloride by providing therapeutic effects that do not have as many shortfalls as currently available formulations. The current formulations have shortfalls associated with them. For example, they do not avoid the first-pass effect, they provide extended-release profiles, they have poor bioavailability, they are not as easy for patients to adhere to due to taste and compliance, and they can produce adverse effects on the gastrointestinal tract due to their release into systemic circulation rather than being targeted for specific areas of the body. We intend to provide better management of CINV using this unique drug delivery method and to improve the health-related QOL of patients receiving chemotherapy (Chua et al., 2020; Mahrous et al., 2021). The high selectivity of Granisetron for the antagonism of the serotonin 5-HT_3_ receptor in the CNS and brain has led to increasing interest for this drug as treatment for nausea and vomiting due to cancer therapy. In most instances, the blocking of serotonin from activating the 5-HT_3_ receptor by Granisetron will alleviate CINV or reduce its occurrence (Gilmore et al., 2018). The effectiveness of Granisetron hydrochloride in preventing and alleviating CINV is well documented, so it is becoming increasingly common to prescribe Granisetron as an adjunctive agent with multiple established Chemotherapy regimens. Granisetron hydrochloride and its limitations are an impediment to its use and user’s positive gratification. Problems with the Granisetron hydrochloride product include unpredictable absorption rates at various doses, a common assortment of side effects associated with dose increases; it has a limited time window for therapeutic efficacy before it will require redosing; it requires someone to either manually/physically administer the medication or the patient must self-administer it, which generates opportunities for noncompliance to occur; and uncertainty surrounding its usefulness can be complex. Therefore, due to these potentially adverse effects on patients and their treatment, there is considerable ongoing interest in creating advanced delivery system designs of ways to improve Granisetron hydrochloride’s effectiveness and/or usefulness as a treatment for patients. As an example of good experimental design is to systematically vary one experimental variable, while controlling the remaining variables, and evaluate how this variance will impact the outcome results of the experiment. This type of a study design typically takes multiple experiments to determine the optimal outcome from the variance; by using design of experiments (DoE), a statistical method for optimization, researchers can quickly identify the factors that impact results with fewer iterations of experimentation (Politis et al., 2017; Anderson and McLean, 2018).

## 2 Materials and methods

### 2.1 Materials

Granisetron hydrochloride was generously donated by MSN Laboratories in Telangana, India. Carbomer (CP934P) was provided by HI media Pvt Ltd., while hydroxy propyl methylcellulose (K4M) was supplied by Loba Chemie Pharmaceuticals, India. Microcrystalline cellulose, sodium alginate (SA), aspartame, talc, and aerosol® 200 were purchased from Sysco research Laboratories Pvt Ltd., India. All remained chemicals employed in this study were of analytical grade and sourced from various suppliers across India.

### 2.2 Formulation optimization using response surface methodology

A randomized 3^2^ central composite design was utilized to study two major formulation factors at 3 levels each and resulted in 11 experimental runs (Table 1). The dependent variables (X_1_) were the amounts of HPMC K4M and carbomer 934P and the dependent variables (Y_1_) were mucoadhesive strength, Y_2_ was time to 50% drug release, and Y_3_ was drug release *In vitro* at 8 h. The statistical analysis of the data was performed with Design-expert software (Version 13.0) and a quadratic polynomial model including the interaction effects was developed from the regression equations generated and the response and contour plots were made as shown in Equation 1.

$$Y=\beta_{0}+\beta_{1}X_{1}+\beta_{2}X_{2}+\beta_{12}X_{1}X_{2}+\beta_{11}X_{12}+\beta_{22}X_{22}$$ (1)

**TABLE 1:** Coded factor for independent variables from Experimental design.

Formulation (s)	Coded variables		Response variables		
	A: HPMC K4M (X_1_)	B: Carbomer (934P) (X_2_)	Adhesive strength (Y_1_)	Time req. For t_50_ percentage drug release (in minutes) (Y_2_)	*In-vitro* percentage drug release at 8 h (Y_3_)
F1	+1	−1	12.37 ± 0.28	380 ± 0.56	61 ± 0.14
F2	−1	0	6.10 ± 0.17	413 ± 0.92	55 ± 0.22
F3	0	0	8.25 ± 0.26	320 ± 0.14	70 ± 1.02
F4	0	+1	10.35 ± 0.31	262 ± 0.23	90 ± 0.36
F5	+1	0	14.17 ± 0.53	277 ± 0.47	89 ± 1.51
F6	0	−1	7.31 ± 0.77	400 ± 0.48	58 ± 1.04
F7	−1	+1	8.17 ± 0.48	364 ± 0.62	62 ± 0.58
F8	0	0	8.25 ± 0.59	325 ± 0.33	72 ± 0.27
F9	0	0	8.45 ± 0.67	330 ± 0.51	70 ± 0.15
F10	−1	−1	5.54 ± 0.98	527 ± 0.44	40 ± 1.30
F11	+1	+1	16.23 ± 0.85	249 ± 0.71	93 ± 0.38

(Mean ± SD; N = 3). Where, +1 Level of X_1_ or X_2_ is higher value, 0 is level of X_1_ or X_2_ is medium value and −1 + 1 Level of X_1_ or X_2_ is Lower value.

In this equation, Y is the response variable, β_0_ is the overall mean response across all observations, and β_1_ is the effect of the independent variables. X_1_ and X_2_ are the effects of each independent variable individually, and the interaction between the two independent variables (X_1_X_2_) represents the change in the response variable caused by changing both independent variables at the same time. By including polynomial terms (X_12_ and X_22_), we can examine whether or not there is nonlinear variation in the relationship. By analyzing both the magnitude and mathematical sign (negative/positive) of the polynomial coefficients, we can draw conclusions (Anderson and Whitcomb, 2016).

### 2.3 Development of granisetron hydrochloride mucoadhesive buccal tablets

Granisetron hydrochloride buccal tablets were formulated by the direct compression method. First, required quantities of the Granisetron hydrochloride, carbomer 934P, HPMC K4M and microcrystalline cellulose were mixed together to properly distribute the drug throughout all of the ingredients. The blends were subsequently combined with aerosil® 200 and talc and mixed for 15 min to create a homogenous mixture. The final preparation was then blended for a further 5 min to enhance the flow properties and compressibility of the mixture prior to tablet compression.

After completion of powder consolidation, tablets were formed by using a single gantry punch tablet machine (Rimek-8, stationary) with a 7 mm round flat punches. To maintain high standards for quality, the final tablet weights and thicknesses were controlled at 200 mg and 4–5 mm, respectively. For the duration of the evaluations, all of the tablets were kept in an air-tight container that is protected from exposure to light.

### 2.4 Characterization of granisetron hydrochloride buccal tablets

#### 2.4.1 FT-IR spectral analysis

**FIGURE 1:**
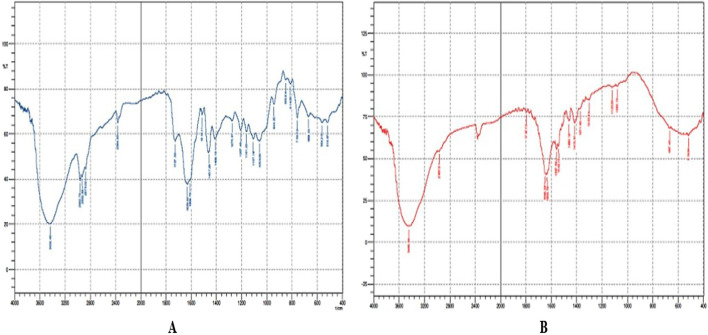
**(A)** FTIR spectrum of Granisetron hydrochloride pure drug, **(B)** Optimized formulation (F3).

To assess compatibility of Granisetron hydrochloride with the selected excipients used in the formulation of the buccal tablet, fourier-transform infrared (FT-IR) spectroscopy (Shimadzu 1205) was employed to collect FT-IR spectra for both pure Granisetron hydrochloride and the physical mixture of Granisetron hydrochloride with its excipients over an appropriate wavelength range. In the UV spectra for pure Granisetron hydrochloride (in Figure 1A), the absorption peaks, which are specific to Granisetron hydrochloride, could be observed. As part of the compatibility testing, the UV spectrum for the drug-excipient was recorded (see Figure 1B) and subsequently compared with that of the pure drug. Interpretation of possible interactions between the drug and its excipients will be based on visual observation of any changes in position and/or intensity of those peaks in the drug and excipient spectra (Zewail et al., 2022).

#### 2.4.2 DSC study of drug–excipient compatibility

To characterize the purified and optimized formulation of Granisetron hydrochloride buccal tablets, single differential scanning calorimetry (DSC) measurements were performed using a Shimadzu DSC-60 (Shimadzu corporation; kyoto, Japan). The aluminum sample holder that holds the sample during testing is maintained under nitrogen gas at a pressure of 20 psi (137.895 kPa) to avoid oxidation. Calibration of the instrument was done with indium metal. A small quantity of Granisetron hydrochloride was added to an aluminum pan, sealed, and tested from 10–200 °C at temperatures increasing by 10° per minute (Abruzzo et al., 2021).

### 2.5 Pre-compressional evaluation of granisetron hydrochloride powder mixture

The bulk density (Db) of a powder is determined by conventional means using a graduated cylinder, filled to 50 mL, with a 10 g sample of powder. The powder should be poured slowly into the graduated measuring cylinder to avoid compressibility and after a 10 g sample of powdered material has been placed in the graduated cylinder, the volume of that sample of powder will be recorded as the volume occupied by that sample of powder; thus, D_b_ can be found from the volume occupied by the sample of powder divided by the weight of the sample of powder (10 g). Tapped density (Dt) was determined utilizing a pharmaceutical tap density tester, Pharma test PT-TD200 (Hainburg, Germany). The powder sample was placed under 1250 taps, with the volume collected at the end of the tapping phase being used to compute the Tap density. For angle of repose (θ), funnel method was used for determination of the angle of repose (θ). The funnel was positioned upright on a flat surface and the powder was discharged through the funnel into a mound shape. The angle between the flat surface and the resulting pile of powder was determined (Kalman, 2021). The properties of the blend powder were measured through Carr’s index and Hausner’s ratio values calculated based upon bulk density and tap density measurements from the established methods as per Equations 2, 3.

$$CI=Dt-\frac{Db}{Dt}*100$$ (2)

$$HR=\frac{Dt}{Db}$$ (3)

### 2.6 Post-compressional evaluation of granisetron buccal tablets

After the compression process, the tablet batches were subjected to evaluation of many physical characteristics, including: tablet thickness, tablet hardness, friability, weight variability, drug content uniformity, degree of swelling index, surface pH, mucoadhesive strength, *In vitro* release rate, release kinetics, and short-term stability of the tablet dosage form (Koradia and Chaudhari, 2018).

From this study, the hardness values of tablets were analyzed with a monsanto hardness tester and reported in kg/cm_2_. The tablets were evaluated for friability (20 Tablets) after removing excess powder using the friabilator, where they were subjected to rotation around the friabilator at 25 rpm for a total of 4 min to determine percentage weight loss during the 4- minute evaluation period to allow the application of the equation to derive the friability percentage value. To determine the surface pH of the tablets after a 1 mL volume of distilled water (pH 6.8 ± 0.05) had been added for swelling the tablets for a 2-h period, a combined glass electrode measuring device was used to take readings after 1 min from time of contact with the tablet. The drug content of the tablets was determined by crushing five tablets into powder form; dissolving the powdered form in a measured volume of distilled water while stirring to dissolve any undissolved particles, then filtering the suspension, and determining UV absorbance (302 nm) of the filtered solution using a blank sample as reference.

The swelling index was determined by recording the initial weight (w_1_) of the buccal tablets placed into 6 mL of a pH 6.8 phosphate buffer at a temperature of 37 °C ± 1 °C. The swelling index was then calculated from the fluctuation of the weights over the time period of 0.5–9 h (w_2_) using Equation 4 (Koirala et al., 2021)

$$\text{Swelling Index}=\frac{\left(W2-W1\right)}{W1}$$ (4)

### 2.7 Assessment of mucoadhesive strength of granisetron buccal tablets

The mucoadhesive strength of the buccal tablet was evaluated using a modified physical balance with bovine cheek pouch as an *In vivo* mucosal model. The mucosa was placed in a 50 mL beaker filled with isotonic phosphate buffer (pH 6.8) maintained at 37 °C. The buccal tablet was attached to a glass stopper and pressed onto the wet mucosa for 3 min with a force of 5 g weights were progressively added to the balance until the tablet was dislodged from the mucosa, with the difference in weight over 5 g being registered as an indication of mucoadhesive strength. For each formulation, three repetitions were conducted to produce an average of the recorded readings; between each application to the mucosa, the tissue was thoroughly rinsed and allowed to rest (Lee et al., 2016).

### 2.8 *In vitro* drug release study of granisetron buccal tablet

The dissolution of a buccal tablet will be tested using a paddle dissolution unit. This study will utilize a paddle dissolution unit set to rotate at 50 rpm, has 200 mL of phosphate buffer (with a pH of 6.8) at 37 °C. A glass disk, shaped at the base of the paddle vessel, holds the buccal tablets in place during testing, and the paddle maintains constant stirring of the tablet during the duration of the testing. Every predetermined time point during testing, 5 mL samples of fluid are removed from the paddle vessel, are filtered through a 0.45 μm filter to remove any remaining solid particles, and the tested paddle vessel then receives a replacement volume of phosphate buffer. The concentration of Granisetron hydrochloride was determined spectrophotometrically at the wavelength of 302 nm and all experiments conducted in duplicate or triplicate (Fan et al., 2020).

### 2.9 Kinetic modeling of granisetron buccal tablet release

The dissolution profiles of Granisetron hydrochloride buccal tablets (F1–F11) were calculated based on the different methods like - zero order, first order, higuchi and korsmeyer-pappas as determined by the DD solver tool. Fit parameters (i.e., adjusted *r* ^2^, akaike information criterion (AIC), and model selection criterion (MSC) were applied to determine the best fitting release kinetics and mechanisms for each formulation, allowing for a more detailed comparative analysis across formulations (Usta and Incecayir, 2022; Askarizadeh et al., 2023).

### 2.10 Stability studies

The Granisetron hydrochloride buccal tablet formulation (F3) has been evaluated for 3 months under accelerated stability testing conditions of 40 °C ± 2 °C and 75% ± 5% RH, using an amber vial with rubber stopper containing fifteen tablets, which were placed in a stability chamber at the above stated conditions throughout the testing period of 3 months. Drug content estimation samples were collected at 1-month intervals throughout the 3-month period and at the end of the 3 months, a dissolution test was undertaken to check the drug release profiles of the tablets under accelerated stability conditions (Celik, 2017).

### 2.11 *In vivo* pharmacokinetic evaluation

The pharmacokinetic evaluation of the drug was conducted in accordance with the CPCSEA guidelines as approved by the IAEC at Vaagdevi Pharmacy College (IEC Approval No. VPC/IAEC/2025/2). Healthy male Wistar rats (200–250 g), maintained under standard laboratory conditions (25 °C ± 2 °C; 55% ± 5% relative humidity; 12-h light/dark cycle) with access to food and water *ad libitum*, and were deprived of food for 24 h before administration. The rats were randomly assigned to one of two test groups (n = 10). The animals in Group I were given a formulated tablet (Kytril®; 1 mg) of Granisetron hydrochloride, prepared using appropriate dispersion methods, while the animals in Group II received the novel formulation (F3) of the therapeutic Granisetron administered as buccal tablets placed in the buccal cavity for adhesion. Blood samples (∼0.5 mL) were collected from the retro-orbital plexus at specified intervals (0–24 h) into heparinized tubes. The plasma obtained following centrifugation was stored at −20 °C until the time of analysis.

Quantification of Granisetron hydrochloride in plasma samples was conducted with a validated HPLC method and pharmacokinetic parameters (C_max_, T_max_, AUC, t½, MRT, clearance, and volume of distribution) were derived from a non-compartmental analysis of the data collected (Kurćubić et al., 2021; Samanthula et al., 2022).

## 3 Results and discussion

### 3.1 Studies on drug–polymer interaction

As shown by FTIR studies, there are no observable signs of any physical/chemical interactions between Granisetron hydrochloride and any of the excipients used in formula preparation. The pure Granisetron hydrochloride IR spectra contained a unique peak at 3105 cm^-1^. This peak represents C-H stretching vibrations which indicates that there are aromatic groups present within the molecule. Another peak was seen at 2864 cm^-1^, and corresponds to C-H stretching vibrations on aliphatic structures.

The characteristic peaks at 1620 cm^-1^ and 1658 cm^-1^ for C=C (aromatic) were present and again confirm the presence of both aromatic and aliphatic groups plus several double bonds found in the structure of Granisetron hydrochloride. These same peaks continue to appear through each formulation IR scans confirming no alteration of drug content within the different formulations and further supporting our conclusion that no drug-excipient interactions were seen.

**FIGURE 2:**
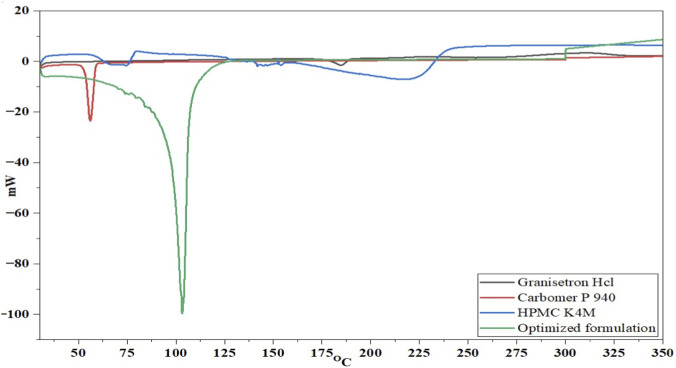
DSC thermogram of Granisetron hydrochloride and optimized formulation (F3).

Figure 2 Provides DSC thermograms for pure Granisetron hydrochloride, several excipients, and actually the optimized formulations. Pure Granisetron hydrochloride has shown its crystalline nature by producing a sharp endothermic peak between 198 °C and 201 °C. Carbomer 940 and HPMC K4M produced characteristic thermal events at 52 °C and 151 °C respectively. However, the optimized buccal tablet formulation, designated F3 produced a broad endothermic peak appearing near 104 °C with no appearance of any defined melting endotherm from the Granisetron hydrochloride. The disappearance of melting endotherm from Granisetron hydrochloride indicates successful molecular dispersion of selected API into the polymeric matrix and confirms that the optimized formulation is compatible and stable as prepared.

### 3.2 Assessment of granisetron hydrochloride physical mixture

Granisetron hydrochloride buccal tablets were manufactured using a direct compression method on the powder blend (also referred to as a core powder) that was used for each formulation, which has been analyzed for pre-formulation characteristics before compression took place. The bulk and tapped densities range from 0.314 ± 0.01 gm/cc to 0.413 ± 0.05 gm/cc and 0.382 ± 0.21 gm/cc to 0.498 ± 0.31 gm/cc, respectively. The compressibility index of each formulation ranged from 10.15 ± 0.41 to 15.34 ± 0.52, the Hausner’s ratio of each formulation ranged from 1.17 ± 0.27 to 1.36 ± 0.41, and angle of repose for each formulation ranged from 20.12 ± 0.06 to 22.50 ± 0.04. This data suggests the powder blends show fair to good flow characteristics and adequate packing capacity. All data is presented in Table 2.

**TABLE 2:** Evaluation of Granisetron hydrochloride physical mixture.

Formulation code	Bulk density gm/cc	True density gm/cc	Carr’s index (%)	Hausner’s ratio	Angle of repose (θ)
F1	0.389 ± 0.02	0.385 ± 0.21	10.15 ± 0.41	1.34 ± 0.07	20.12 ± 0.07
F2	0.371 ± 0.04	0.388 ± 0.25	11.21 ± 0.07	1.25 ± 0.06	21.31 ± 0.05
F3	0.338 ± 0.03	0.384 ± 0.23	11.37 ± 0.42	1.23 ± 0.34	21.34 ± 0.06
F4	0.319 ± 0.07	0.391 ± 0.24	12.38 ± 0.15	1.31 ± 0.27	20.27 ± 0.01
F5	0.314 ± 0.01	0.397 ± 0.28	11.84 ± 0.48	1.27 ± 0.18	20.12 ± 0.06
F6	0.327 ± 0.06	0.383 ± 0.22	10.64 ± 0.51	1.34 ± 0.29	20.42 ± 0.05
F7	0.358 ± 0.02	0.498 ± 0.31	13.17 ± 0.54	1.36 ± 0.41	20.17 ± 0.03
F8	0.333 ± 0.08	0.390 ± 0.21	15.34 ± 0.52	1.33 ± 0.31	21.74 ± 0.03
F9	0.337 ± 0.06	0.382 ± 0.21	12.53 ± 0.84	1.32 ± 0.23	21.42 ± 0.08
F10	0.342 ± 0.04	0.393 ± 0.23	13.64 ± 0.72	1.32 ± 0.25	21.37 ± 0.03
F11	0.326 ± 0.03	0.384 ± 0.26	11.86 ± 0.07	1.32 ± 0.37	20.42 ± 0.08

(Mean ± SD; n = 3).

### 3.3 Post-compression evaluation of granisetron buccal tablets

All batches of Granisetron hydrochloride buccal tablets were manufactured with a complete formulation of 200 mg of active pharmaceutical ingredient (API). The average weight of the prepared batches ranged from 200.03 ± 1.25 mg to 203.17 ± 0.29 mg and was consistent across all batches. The thickness of the buccal tablets followed the same trend with the range of 4.35 ± 0.03 mm to 4.79 ± 0.04 mm being recorded for average thickness and hardness ranging from 4.74 ± 0.28 to 5.62 ± 1.45 kg/cm^2^. The surface pH for the core layer of the Granisetron buccal tablets was observed to fall within a pH range of 6.53 ± 0.05 and 7.21 ± 0.06. All of the batches were tested for friability and the results were determined to be below 1% w/w which was in compliance with the limits set forth in the pharmacopeia. The percentage drug content for all batches was found to fall within the range of 98.18% ± 0.51% to 99.64% ± 0.74%. Table 3 contains all of the post-compression test results.

**TABLE 3:** Evaluation of Granisetron hydrochloride buccal tablets F1-F11.

Code	Tablet Thickness (nm)	Hardness of tablet (kg/cm2)	Friability (%)	Wt. Variation (mg)	Surface pH (pH)	Swelling index (%)	Drug content (%)
F1	4.41 ± 0.04	4.74 ± 0.28	0.53 ± 0.04	200.03 ± 1.25	6.57 ± 0.04	82.34 ± 0.25	98.57 ± 0.52
F2	4.35 ± 0.03	4.89 ± 1.37	0.61 ± 0.04	201.04 ± 1.25	7.14 ± 0.03	92.68 ± 0.31	99.64 ± 0.74
F3	4.51 ± 0.04	4.96 ± 1.41	0.67 ± 0.02	200.05 ± 1.21	6.53 ± 0.05	90.36 ± 0.53	99.26 ± 1.24
F4	4.45 ± 0.01	5.21 ± 0.28	0.71 ± 0.01	201.08 ± 0.08	7.21 ± 0.03	84.29 ± 0.51	99.28 ± 0.55
F5	4.52 ± 0.05	5.03 ± 0.85	0.54 ± 0.03	203.17 ± 0.29	6.59 ± 0.02	80.26 ± 0.28	98.63 ± 0.59
F6	4.61 ± 0.06	5.51 ± 2.14	0.57 ± 0.05	202.15 ± 0.87	7.21 ± 0.06	92.64 ± 0.39	99.32 ± 0.58
F7	4.79 ± 0.04	5.34 ± 0.19	0.72 ± 0.07	200.08 ± 0.08	6.64 ± 0.01	88.98 ± 0.45	99.53 ± 0.67
F8	4.68 ± 0.07	5.28 ± 0.25	0.68 ± 0.05	201.28 ± 0.06	6.59 ± 0.05	89.67 ± 0.58	98.62 ± 0.64
F9	4.48 ± 0.01	5.42 ± 1.08	0.74 ± 0.05	202.04 ± 0.84	6.71 ± 0.02	91.38 ± 0.19	98.18 ± 0.51
F10	4.49 ± 0.04	5.62 ± 1.45	0.68 ± 0.03	202.03 ± 1.18	6.70 ± 0.04	91.89 ± 0.27	99.64 ± 0.74
F11	4.41 ± 0.02	5.36 ± 1.27	0.58 ± 0.06	203.03 ± 0.96	6.53 ± 0.05	78.35 ± 0.24	98.58 ± 0.17

(Mean SD ± n = 3).

### 3.4 *In-vitro* dissolution profile of granisetron buccal tablets (F1–F11)

**FIGURE 3:**
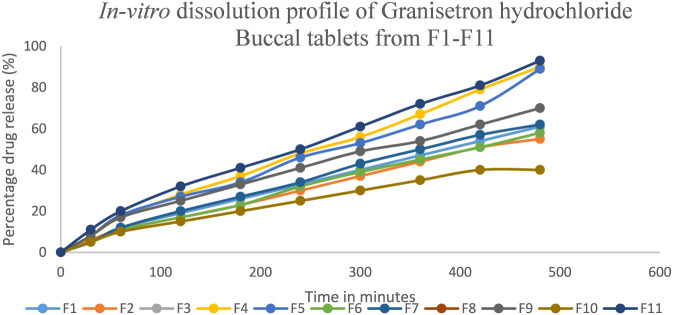
*In-vitro* dissolution profile Granisetron hydrochloride buccal tablets (F1-F11).

The authors conducted a study on how the release of the drug form of Granisetron hydrochloride through buccal tablets (F1-F11) occurred over an 8-h period. In this study, the amount of drug released from the tablets with the lowest amount (F3) was reported to be 40.78 ± 1.27, while the highest reported release (F11) was 93.57 ± 0.85. The variation in drug release amounts can be attributed to the differences between two manufacturing materials: hydroxyl propyl methylcellulose (HPMC K4M) and carbomer 934P as demonstrated in Figure 3. In addition to this study has further demonstrated that hydroxyl propyl methylcellulose is a unique polymeric material that has characteristics such as high viscosity (which results in thickening), gelling up in the mouth and also assisting in triple-hydration, among other beneficial effects for the pharmaceutical industry. The swelling and erosion properties of HPMC will have a significant effect on the release of the drug from the mouth to the buccal cavity. The role of carbomer in drug release is an important factor in a drug’s ability to be released out of the tablet.

The hydrophilicity of carbomer increases its ability to swell, which extends the time the medicated tablet will remain in contact with the buccal area and impacts on the rate of drug release from the tablet. The swelling and subsequent change to the structure of the tablet resulting from the use of carbomer may also ultimately influence the overall DE-80 buccal tablet release profile.

Both HPMC and carbomer are necessary components that aid in the disintegration of Granisetron buccal tablets. The properties associated with HPMC (e.g., its thickness and its ability to create gels) and carbomer (e.g., its water absorption ability and swelled/gell forming ability) will be predominant variables in determining how quickly Granisetron will dissolve when taken under the tongue.

### 3.5 Optimization of granisetron buccal tablets using response surface method

#### 3.5.1 Mucoadhesion strength (Y_1_)

The mucoadhesive strength of granisetron buccal formulations (F1 to F11) exhibited a wide range of values for the measured mucoadhesive strength (Y_1_), which ranged from 6.17 ± 0.58 g to 16.23 ± 0.48 g. Thus, the amount of mucoadhesives employed is of major significance to the mucoadhesive robustness of the final buccal formulations. A quadratic regression model was utilized to study the effect of the mucoadhesive concentration on the mucoadhesive strength. The regression equation for the quadratic regression model is presented in Equation 5

$$\text{Mucoadhesion strength }\left(Y_{1}\right)=+28.90-5.34X_{1}-6.57X_{2}+10.28X_{12}+12.36X_{1}^{2}+6.86X_{2}^{2}$$ (5)

**FIGURE 4:**
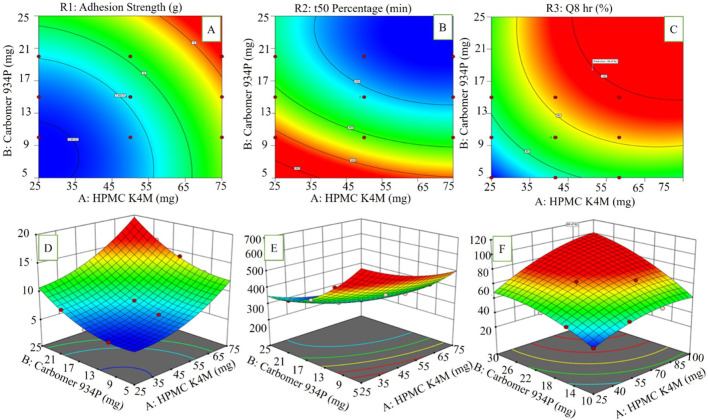
Response surface methodology (RSM) plots illustrating the effect of formulation variables on drug release parameters: **(A)** 2D contour plot and **(D)** 3D surface plot for percentage drug release (Q) at 8 h; **(B)** 2D contour plot and **(E)** 3D surface plot for t_50_ (time for 50% drug release); **(C)** 2D contour plot and **(F)** 3D surface plot for percentage drug release (Q) at 8 h.

An analysis of variance (ANOVA) showed that the model is statistically significant, *r* ^2^ = 0.998, *p < 0.001*. The individual components (X_1_ HPMC K4M & X_2_ carbomer 934P) were found to have a negative impact on adhesion to mucus of test formulations; however, when combined together, their interaction created an increased mucosal adhesion coefficient of (+10.28), thereby providing a greater degree of adhesion to mucus than either of the individual components alone. This demonstrates that by increasing the concentration of both polymers, it is possible to obtain greater mucoadhesive strength. Additionally, the use of HPMC K4M (X_1_) and carbomer 934P (X_2_) provides for improved control of drug release and optimal release kinetics. Figures 4A,D demonstrate that the design of HPMC K4M and carbomer 934P created a synergistic interaction that resulted in an increase in the rate and efficiency of the mucoadhesiveness of the buccal tablets.

#### 3.5.2 Time required for (t_50_ percentage) drug release (Y_2_)

This study evaluated how the HPMC K4M (X_1_) and carbomer 934P (X_2_) are related to the time to reach 50% of drug release (t_50_, Y_2_) in relation to Granisetron buccal tablet formulations (F1-F11). The t_50_ values ranged between 249 ± 0.27 to 413 ± 0.23 min (time to reach 50% release), which illustrates the impact of mucoadhesive polymers upon the release rate of the drug. To determine the relationship between t_50_ and the two variables HPMC K4M and carbomer 934P, a quadratic regression model was fitted. The regression equation is shown in Equation 6.

$$t_{50}\;\%\text{ drug release }\left(Y_{2}\right)=+35.28-6.12X_{1}-3.34X_{2}-18.27X_{12}+2.14X_{1}^{2}+3.43X_{2}^{2}$$ (6)

The ANOVA analysis demonstrated that the data fits well with a high coefficient of determination (*r* ^2^ = 0.992) and statistical significance (*p < 0.0001*); thus, it can be successfully utilized. In addition, upon creating the polynomial equation to represent the interaction of HPMC K4M and carbomer 934P (X_1_X_2_) there was a significantly higher negative interaction effect present due to these two polymers to release the drug, a coefficient of −18.27 than using either polymer alone. Carbomer, when hydrated, forms a gel which assists in mucoadhesion and allows for greater adhesion to buccal mucosae, while HPMC K4M forms a viscous gel that controls drug diffusion rate providing for sustained drug release through the polymer’s continued diffusion ability from its gel matric.

#### 3.5.3 Percentage drug release at Q8hr (Y_3_)

After 8 h (Y_3_), the % occurrence of a drug being released was in the range of 40.78% ± 1.27% to 93.57% ± 0.85% for every formulation (F1-F11). Using statistical analysis, there was indication that the differences among various formulations were also attributed to the choices and volume of bio adhesive polymers used for the respective products. The statistical data were evaluated by quadratic regression, as displayed in Equation 7.

$$\%\text{ drug release }Q\text{ at }8\text{hr }\left(Y3\right)=+22.24+3.96X_{1}+4.64X_{2}+25.89X_{12}-1.35X_{1}{\text{​}}^{2}-2.57X_{2}{\text{​}}^{2}$$ (7)

Results from ANOVA support that there was an excellent fit of a polynomial regression model to the data, with *r* ^2^ = 0.952 and *p-value of 0.002 (p < 0.05)*. Therefore, good regression modelling of the statistical data with a polynomial regression function. According to the polynomial regression equation, the release of a drug from each formulation is significantly affected by a multiple interaction between the two polymers, HPMC K4M(X_1_) and carbomer 934P (X_2_). The collective release of drug from both polymers was significantly higher than the release of drug through either polymer. Additionally, the interaction coefficient of 25.74 of HPMC K4M(X_1_) and carbomer 934P (X_2_) together illustrate that increasing both polymers’ concentrations will exponentially increase the percentage of drug released from the tested formulations after 8 hours. The mutual benefit of the two polymers’ interactions is further highlighted in Figures 4C,F where both of the polymers have shown the same and positive effects on the percentage of drug released from their respective formulations.

### 3.6 Selection of optimized batch based on desirability approach

**FIGURE 5:**
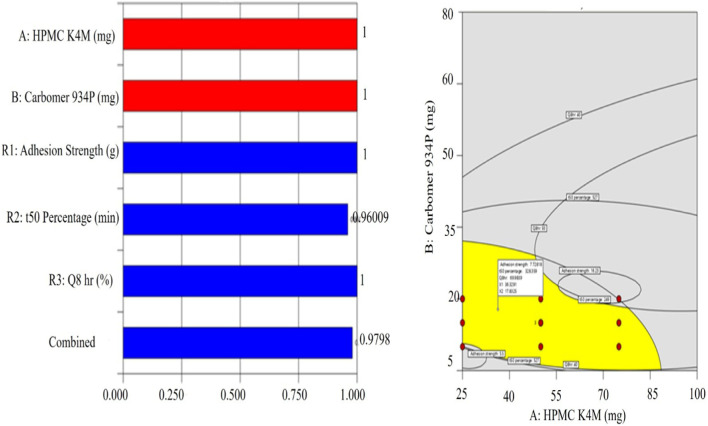
**(A)** Desirability plot for optimized batch, **(B)** Overlay plot for optimized batch F3.

The optimal formulation was determined through the application of the desirability function using the software Design-Expert®, which enables the combination of multiple response variables into one score from 0 to 1, where the closer the score is to 0, the worse the performance; conversely, the higher the score is to 1, the better the performance. In this way, F1-F11 were assessed for applicability in the preparation of buccal tablets of Granisetron hydrochloride. The desirability and overlay plots from the software are illustrated in Figure 5. F3 was the most favorable formulation with a desirability score of 0.980 out of 1.00 (Figure 5A), whereas the overlay chart (Figure 5B), reveals the configurational area that encompassed the intersection of all desired response factors. The formulation of F3 was found to possess the highest mucosal adhesion (8.63), the t_50_ time of release at 299.4 min, and a drug release percentage of over 70% at the time of 8 h. In summary, the analysis completed by Design-Expert® using the desirability score indicated that HPMC K4M and carbomer 934P would be appropriate functional components of the formulation, and that specific ranges of concentration for both agents must be used to produce the predetermined associated performance characteristics of the product.

### 3.7 Assessment of drug release kinetics mechanism

The DD solver software was used to analyze the drug release profiles for the buccal tablets containing Granisetron hydrochloride using the 11 different formulations (F1 – F11) to determine the drug’s release kinetics by comparing the various mathematical models that can be applied to a drug’s release profile and the results of these comparisons for each formulation are in Table 4. Among all the formulations, formulation F3 had the highest correlation coefficients across all models. Specifically, for the zero-order model, the correlation coefficient (*r* ^2^) = 0.9856, AIC = 33.17 and MSC = 3.941 were the best fit for this formulation. Analysis by the first-order model also had a correlation coefficient (*r* ^2^) = 0.9817, an AIC value of 29.37 and the best MSC fit for F3 (4.962). The Higuchi model analysis had a high correlation between fitted and experimental release data (*r* ^2^ = 0.9895, AIC = 34.28, MSC = 5.275) which suggests that this model can be used to predict release from Granisetron hydrochloride buccal tablets. Hixson–Crowell model analysis produced excellent fitting parameters for F3, including correlation coefficient (*r* ^2^) = 0.9951, AIC = 37.21, and MSC = 5.374. The Korsmeyer–Peppas model suggested that the F3 formulation would produce a satisfactory fit (*r* ^2^ = 0.9917, AIC = 28.64, and MSC = 6.745). The release exponent (n = 0.833) indicates that the mechanism of drug release from formulation F3 follows non-Fickian diffusion.

**TABLE 4:** Summary of optimized formulation (F3) from DD solver software.

Release order parameters	Zero order (k_0_)	First order (k_1_)	Higuci model (k_H_)	Hixon-Crowell (k_HC_)	Korsmeyer-peppas (k_KP_)	n
*R* ^2^_adjusted_	0.9856	0.9817	0.9895	0.9951	0.9971	0.833 (non-fickian diffusion)
*R* ^2^	0.9856	0.9817	0.9895	0.9858	0.9967	
AIC	33.17	29.37	34.28	37.21	28.64	
MSC	3.941	4.962	5.275	5.374	6.745	

Where, *r* ^2^ is the regression coefficient, Akaike Information Criterion (AIC), model selection criteria etc.

Using a number of mathematical models to investigate the release kinetics and underlying mechanisms of the different Granisetron hydrochloride formulations, this research assessed how effectively Granisetron hydrochloride formulations will release Granisetron hydrochloride from the formulations. The kinetics provide insight into how the release kinetics of the formulations change over time. Stability testing under relevant time frame (3 months) at controlled conditions demonstrated for the optimized formulation (F3) demonstrated physicochemical stability. The dissolution profile for F3 was found to be very similar to the dissolution profile for Kytril® 1 mg (Granisetron hydrochloride), which is a commercial formulation of Granisetron. Using the similarity factor *(f*_*2*_ *)* to compare the dissolution profiles, it was found that the dissolution profiles for F3 and Kytril® 1 mg *(f*_*2*_ *= 79)* were very similar.

### 3.8 *In-vivo* pharmacokinetic evaluation studies

**FIGURE 6:**
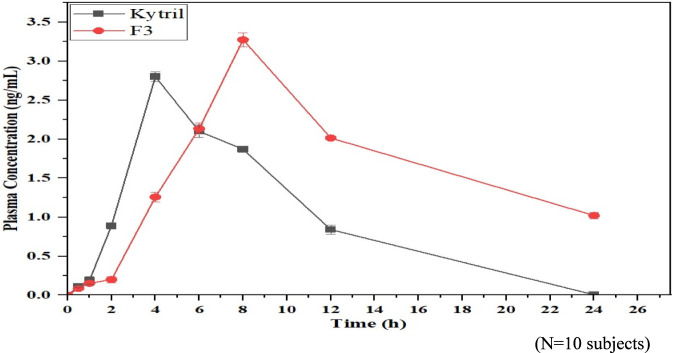
Mean plasma concentration- Time profile of marketed formulation (Kytril) and optimized formulation (F3).

The study determined that F3 (formulation) demonstrated the best performance characteristics for its *In vitro* mucoadhesive adherence, its kinetics of release, and dissolution characteristics. Formulation F3 had a satisfactory mucoadhesive strength of 8.25 ± 0.12 g, a t_50_ value of 320 ± 0.35 min, and after 8 h, the cumulative percentage of the drug released was 70.23 ± 2.14 percent. This formulation was then further investigated for how it behaved *In vivo* through the pharmacokinetics study. This is shown in Figure 6, which revealed that compared to the oral conventional formulation (Kytril® 1 mg), the buccal formulation F3 had much delayed absorption a T_max_ of 8.0 h compared with 4.0 h due to a prolonged period of time for drug to be released. The maximum concentration (C_max_) that was obtained from the buccal formulation (F3) was 3.485 ± 2.35 ng/mL as compared to the oral formulation 2.358 ± 1.38 ng/mL). Furthermore, the buccal delivery of F3 resulted in a significant amount more of systemic exposure to the drug when compared with the oral formulation with respect to AUC_0_–t and AUC –0∞ , with the values being about 5–6 times greater *(p < 0.04).*

Results from one-way ANOVA indicate that there is a statistically significant difference in the area under the concentration vs. time curve (AUC) between the oral conventional tablet form and the buccal formulated product *(p < 0.04),* which can be found in Table 5. With buccal administration of formulation F3, the bioavailability was increased compared to oral doses by about 5–6 times. This increase in bioavailability can most likely be ascribed to the prolonged residence time of the drug within the buccal mucosa, the controlled release of the drug from the polymer matrix, and partial bypass of the hepatic first-pass metabolism.

**TABLE 5:** Pharmacokinetic parameters of Granisetron buccal tablets (F3) and Marketed formulation.

Parameters	Units	F3	Kytril
Lambda_z	1/h	0.051225237	0.021461871
t1/2	h	8.53136106	4.29667975
Tmax	h	8	4
Cmax	ng/mL	3.485	2.3
Tlag	h	0	0
Clast_obs/Cmax	​	0.442180775	0.041304348
AUC 0-t	ng/mL*h	53.882	10.8375
AUC 0-inf_obs	ng/mL*h	83.96482796	15.26395467
AUC 0-t/0-inf_obs	​	0.641721079	0.710006039
AUMC 0-inf_obs	ng/mL*h^2^	1942.892383	374.5622908
MRT 0-inf_obs	h	23.13936001	24.53900701
Vz/F_obs	(mg)/(ng/mL)	191.8105839	2518.368607
Cl/F_obs	(mg)/(ng/mL)/h	9.825542671	54.04890264

(N = 10 subjects).

Formulation F3 (optimized buccal tablet) exhibited a delay in T_max_ relative to marketed oral product Kytril, marked by an increased C_max_, as well as a significantly increased AUC, reflective of an improvement in the duration of systemic exposure as well as the total amount of drug that enters the systemic circulation (Figure 6). Compared to the oral route, buccal route delivers benefits for managing nausea and vomiting due to chemotherapy, reducing GI variability and providing prolonged systemic concentrations. Overall, formulation F3 will likely provide a superior pharmacokinetic profile, and would be a viable alternative to conventional oral administration.

While these findings appear promising, more research needs to be conducted before being used clinically. Several necessary studies will be assessing long-term safety and local mucosal tolerance; conducting scale-up and stability testing as directed by regulatory agencies; and performing human pharmacokinetic and bioequivalence studies. Ultimately, well-executed clinical trials will be necessary to establish the safety, efficacy and acceptability in patients of the clinical use of the optimized buccal formulation.

## 4 Conclusion

The buccal tablets of Granisetron hydrochloride have been formulated with optimal amounts of HPMC K4M and carbomer 934P, both of which are biodegradable polymers. By optimizing the concentration of these two polymers, a greater ability for the tablets to dissolve quickly with the use of the polymers and the ability to provide a gradual, long-lasting release of Granisetron can be achieved. The effects of HPMC K4M and carbomer 934P on mucoadhesive strength (Y_1_), time to attain 50% drug released (t_50_, Y_2_) and percentage of drug released after 8 hours (Q_8_, Y_3_) were statistically optimized using Design-Expert® *via* the response surface methodology. The F3 formulation, identified as the optimal preparation, achieved compliance with the IP. Further analysis of the *In-vitro* dissolution data of F3 using DDsolver resulted in the determination of drug-release kinetics, the drug-release mechanisms and the degree of similarity *(f*_*2*_ *).* The results of the pharmacokinetic testing showed there were statistically significant differences (p < 0.04) between the C_max_, T_max_ and AUC values of the buccal formulation and those of the standard oral Granisetron formulation. The construction of sustained-release obtained by the F3 formulation demonstrate the potential effectiveness of this formulation as sustained-release therapeutics.

## Data Availability

The original contributions presented in the study are included in the article/supplementary material, further inquiries can be directed to the corresponding author.
